# Influence of Ovophospholipids on Lymphocyte Subsets and Humoral Immune Response in Mice

**DOI:** 10.3390/molecules30112253

**Published:** 2025-05-22

**Authors:** Magdalena Lis, Marianna Szczypka, Agnieszka Suszko-Pawłowska, Aleksandra Pawlak, Łukasz Bobak, Bożena Obmińska-Mrukowicz

**Affiliations:** 1 Department of Pharmacology and Toxicology, Faculty of Veterinary Medicine, Wroclaw University of Environmental and Life Sciences, C. K. Norwida 31, 50-375 Wrocław, Poland; marianna.szczypka@upwr.edu.pl (M.S.); agnieszka.suszko@upwr.edu.pl (A.S.-P.); aleksandra.pawlak@upwr.edu.pl (A.P.); bozena.obminska-mrukowicz@upwr.edu.pl (B.O.-M.); 2 Department of Functional Food Products Development, Faculty of Biotechnology and Food Science, Wroclaw University of Environmental and Life Sciences, Chełmońskiego 37, 51-630 Wrocław, Poland; lukasz.bobak@upwr.edu.pl

**Keywords:** ovophospholipids, phospholipids, lymphocytes, humoral immune response

## Abstract

Designed hen eggs enriched in DHA and EPA are an alternative source of essential phospholipids. This study assessed the effects of ovophospholipids on lymphocyte subsets in non-immunized mice and on the humoral immune response in sheep red blood cells (SRBC)-immunized mice. Ovophospholipids were administered orally for 14 days (once a day) at doses of 100, 10, and 1 mg/kg. Ovophospholipids increased the total lymphocyte count of in the lymphoid organs. At 10 and 1 mg/kg, ovophospholipids increased the subsets of CD4^−^CD8^−^ and CD4^+^CD8^+^ thymocytes but decreased the percentage of CD4^+^ and CD8^+^ thymocytes. A stimulating effect on splenocytes was particularly evident 24 h after administration of the 10 and 1 mg/kg doses. Ovophospholipids also elevated the absolute counts of CD3^+^ and CD19^+^ splenocytes. An increase in the absolute count of CD3^+^, CD4^+^, CD8^+^, and CD19^+^ lymphocytes of the mesenteric lymph nodes was observed 24 h after administration of the lowest dose. The increase in the percentage and absolute count of CD19^+^ cells and in the absolute count of CD3^+^ cells was still observed after 72 h. At all doses, ovophospholipids elevated the number of plaque-forming cells on day 4 and increased 2-mercaptoethanol-resistant antibody titer on day 7 after priming. In conclusion, ovophospholipids can modulate the immune response in mice.

## 1. Introduction

Phospholipids belong to complex lipids that are one of the major components of cell membranes, playing an essential role in the proper functioning of the human and animal body. The richest natural sources of phospholipids are nervous tissue, liver, egg yolk, and the seeds of oil-producing plants, especially soybeans and rapeseed [1,2,3,4].

Currently, oil plants, particularly soya, are the main industrial source of phospholipids. However, the phospholipid composition of plant-derived sources differs from that of hen egg yolk. The oil plants, especially soya, contain about 2% of phospholipids [1,4]. Meanwhile, the yolk of a typical egg contains 33% of phospholipids in its dry matter, of which pure phosphatidylcholine (lecithin) accounts for 76% [1,3].

Omega-3 polyunsaturated fatty acids (n-3 PUFAs) (phospholipid components esterified in the phospholipid structure) play an important role in maintaining the human/animal body homeostasis, being involved in many physiological processes. Improper diet, rich in saturated fatty acids and poor in n-3 PUFAs, with inappropriate n-6/n-3 PUFA ratio is, among others, a reason behind many the so-called “lifestyle diseases” [1]. The most relevant long-chain n-3 PUFAs are docosahexaenoic acid (DHA), eicosapentaenoic acid (EPA), and α-linolenic acid (ALA). The main sources of DHA and EPA are marine fish oil and algae. In phospholipids of plant origin, the only representative of n-3 PUFAs is ALA. In contrast, yolk phospholipids include a wider palette of n-3 PUFAs, such as ALA or DHA. It has been proven that the phospholipid content of egg yolk depends on genetic factors, the age and living conditions of laying hens, and, importantly, the type of feed they receive [5]. Therefore, it is possible to change the phospholipid profile of an egg yolk through introduction of some modifications in the hen diet, i.e., supplementation with ingredients rich in target fatty acids (peat preparations, rapeseed oil, linseed oil, sea algae) [1]. Such eggs, called designed eggs, may contain many more long-chain n-3 PUFAs (in comparison with ordinary eggs), also including EPA [1].

Taking into account the need to increase dietary intake of n-3 PUFAs (especially DHA and EPA) by humans and some limitations regarding edible products (sensory features—fish taste and smell, intolerance to soya protein) in which they are found, searching for their alternative sources is of special importance. Ovophospholipids isolated from designed eggs enriched in DHA and EPA seem a good solution.

The immunomodulatory action of DHA or EPA has been confirmed in many studies.

Regarding the impact of n-3 PUFAs on cytokine (IL-1β, IL-4, IL-6, IL-10, TNF-α, IFN-γ) synthesis, the results vary depending on the study. A stimulating effect of n3-PUFA on lymphocyte proliferation and cytokine production was observed by some authors [6], but not by others [7]. Downregulation of pathologically raised cytokine levels was also reported [8,9,10].

EPA and DHA reduced in vitro proliferation of concanavalin A (ConA)-stimulated T lymphocytes isolated from blood of both healthy and type I diabetic people, decreased IL-2 production, and increased IL-4 secretion by these cells [11]. Chapkin et al. [12] demonstrated ex vivo a suppressive effect of DHA on the proliferative response of purified murine T lymphocytes, following a direct stimulation of T cell receptor and coreceptor αCD3/αCD28. An in vivo study on an experimental autoimmune encephalomyelitis model showed significant inhibition of proliferation and IFN-γ and IL-17 production by CD4^+^ T cells from the spleen and CNS, isolated from mice fed with DHA-enriched diet [13].

The effect of n-3 PUFAs on regulatory T cells (Treg) was also investigated. It was shown [14] that long-term administration of n-3 PUFAs to mice enhanced the percentage of hepatic Tregs as well as up-regulated the expression of IL-10, PPAR-γ (peroxisome proliferator activated receptor gamma), and TGF-β (transforming growth factor β). DHA, administered to mice, alleviated atopic dermatitis by inducing the differentiation of CD4^+^Foxp3^+^ Tregs [15]. Dendritic cells (DCs) cultured with DHA decreased T-cell proliferation and raised the proportion of T cells expressing Foxp3, indicating that DHA can promote induction of Tregs [16]. Splenic CD4^+^ T cells isolated from C57BL/6 mice fed with diets enriched with DHA and/or EPA showed reduced polarization into inflammatory Th17 cells but not into Treg cells [17].

Gorjao et al. [6] showed that supplementation of DHA and EPA elevated phagocytic activity of neutrophils and monocytes, as well as the chemotactic response and production of reactive oxygen metabolites by neutrophils. On the other hand, Kew et al. [7] demonstrated no effect on the function (phagocytosis and the expression of adhesion molecules) of neutrophils and monocytes.

As shown in the studies cited above and others [18,19,20,21,22,23,24,25,26], the results regarding many aspects of the immune response are inconsistent and therefore inconclusive. For this reason, there is still a need for further investigation in this area.

The aim of this study was to determine the effect of ovophospholipids isolated from designed eggs enriched with n-3 PUFAs on lymphocyte subsets in the thymus, spleen, and mesenteric lymph nodes in non-immunized mice and on humoral immune response in SRBC-immunized mice, depending on the applied dose.

## 2. Results

### 2.1. Effects of Ovophospholipids on Lymphocyte Subsets

**Figure 1:**
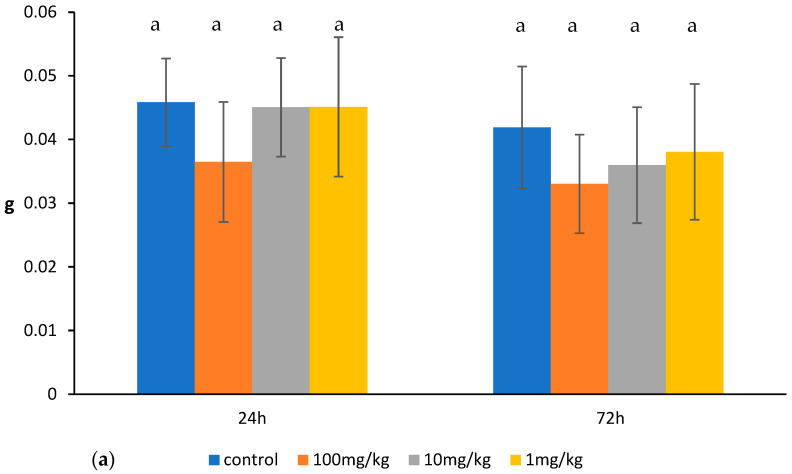

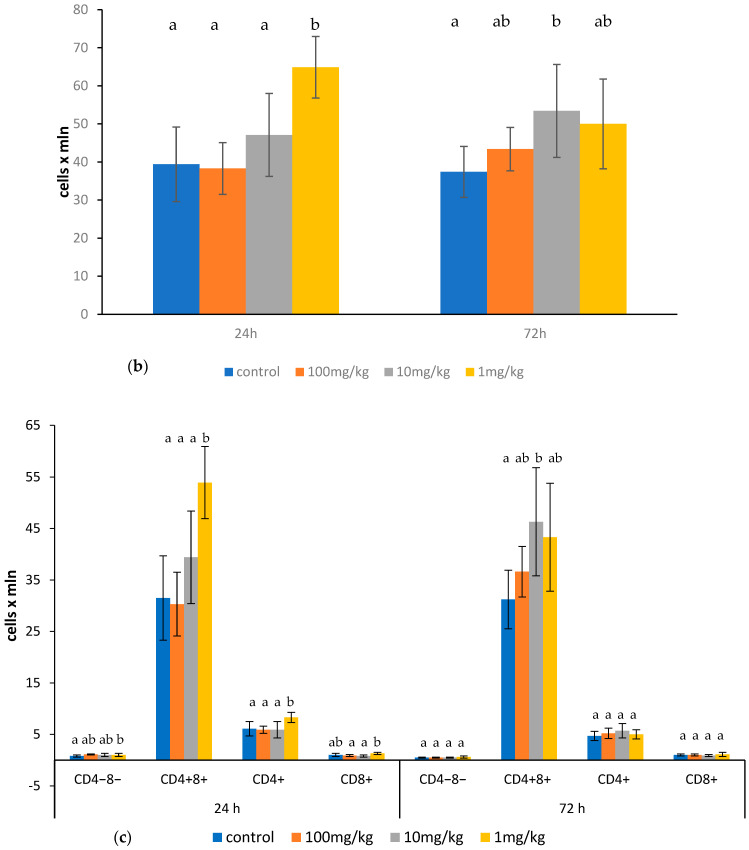

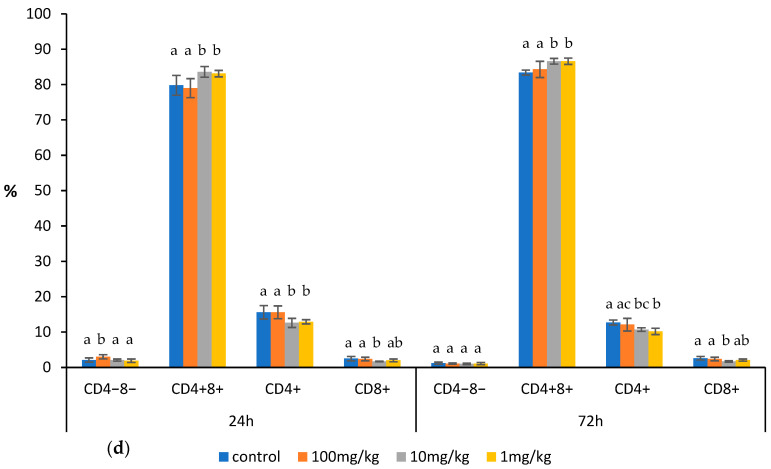
Thymus weight (**a**), total count of thymocytes (**b**), absolute count of thymocyte subpopulations (**c**), and the percentage of thymocyte subpopulations (**d**) in mice treated for 14 days (once a day) with ovophospholipids. The mean value (*n* = 7) and standard deviations are shown. Data with different superscript letters differ significantly (*p* < 0.05).

Ovophospholipids administered at a dose of 10 mg/kg increased the percentage of double-positive CD4^+^CD8^+^ thymocytes with corresponding decrease in the percentage of single-positive CD4^+^ and CD8^+^ thymic cells 24 h after their last administration. Seventy-two hours after administration of the last dose of 10 mg/kg, an increase was noticed in the total count of thymocytes and the percentage and absolute count of CD4^+^CD8^+^ thymocytes, accompanied by a decrease in the percentage of CD4^+^ and CD8^+^ thymic cells. The impact of a 1 mg/kg dose on the thymocytes was noticed especially 24 h after its last administration. Ovophospholipids enhanced the total count of the thymocytes as well as absolute count of CD4^−^CD8^−^ and CD4^+^ thymic cells (but decreased their percentage) and the percentage and absolute count of CD4^+^CD8^+^ thymocytes. The increase in the percentage of CD4^+^CD8^+^ and decrease in the percentage of CD4^+^ thymocytes 72 h after the last dose was observed. The only change noticed after administration of 100 mg/kg ovophospholipids was an increase in the percentage of CD4^−^CD8^−^ thymic cells that was observed 24 h after the last dose (Figure 1b–d). Ovophospholipids at the examined doses did not influence the thymus weight (Figure 1a).

**Figure 2:**
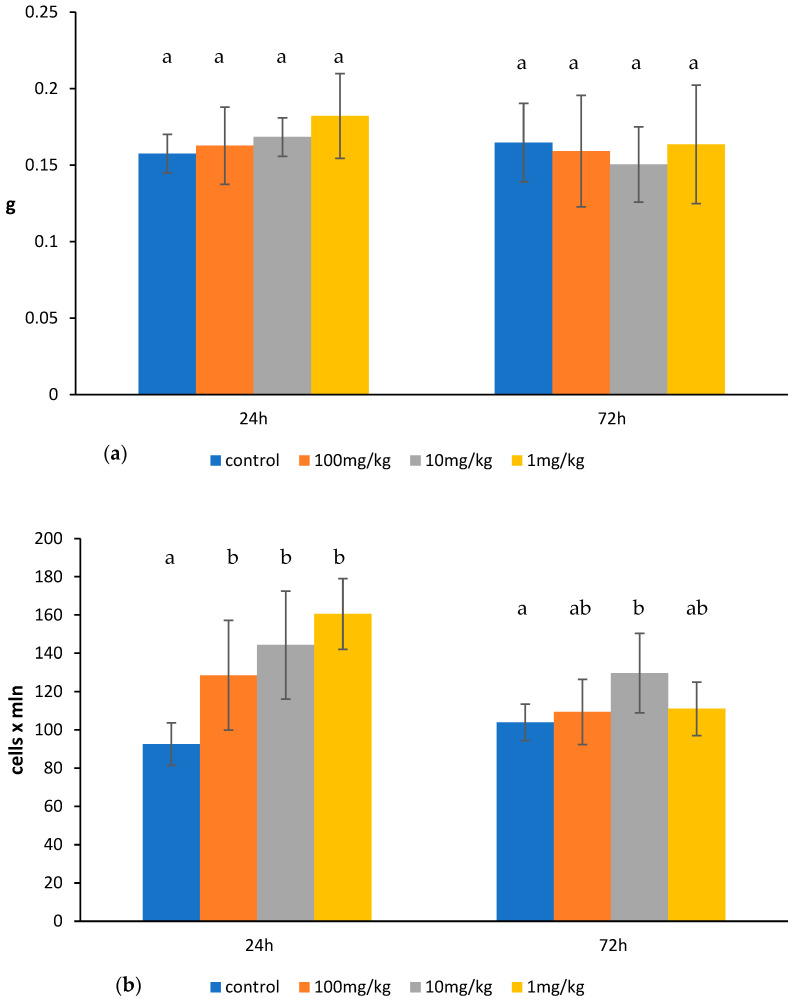

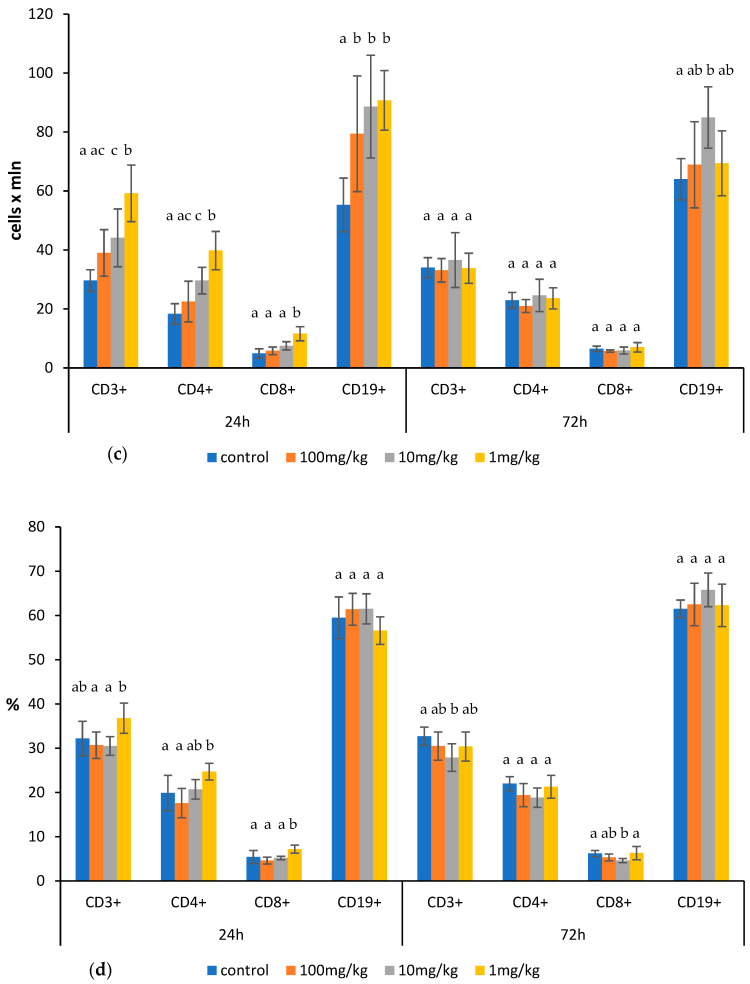
Spleen weight (**a**), total count of splenocytes (**b**), absolute count of splenocyte subpopulations (**c**), and the percentage of splenocyte subpopulations (**d**) in mice treated for 14 days (once a day) with ovophospholipids. The mean value (*n* = 7) and standard deviations are shown. Data with different superscript letters differ significantly (*p* < 0.05).

Ovophospholipids exerted a stimulating effect on the splenic lymphocytes. The effect was observed especially after 24 h. At that time, ovophospholipids elevated the total count of the splenocytes in a dose-independent manner. A rise was noticed in the absolute count of the CD3^+^ and CD4^+^ splenic lymphocytes after the administration of 10 and 1 mg/kg doses of ovophospholipids. An increase in the percentage of CD4^+^ splenocytes and the percentage and absolute count of CD8^+^ cells in the presence of 1 mg/kg of ovophospholipids was also seen. Ovophospholipids elevated the absolute count of B lymphocytes at all examined doses. Seventy-two hours after the last dose of 10 mg/kg, a rise in total count but a decrease in the percentage of CD3^+^ and CD8^+^, and an increase in the absolute count of CD19^+^ splenocytes were found (Figure 2b–d). Regardless of the dose, ovophospholipids did not change the spleen weight (Figure 2a).

**Figure 3:**
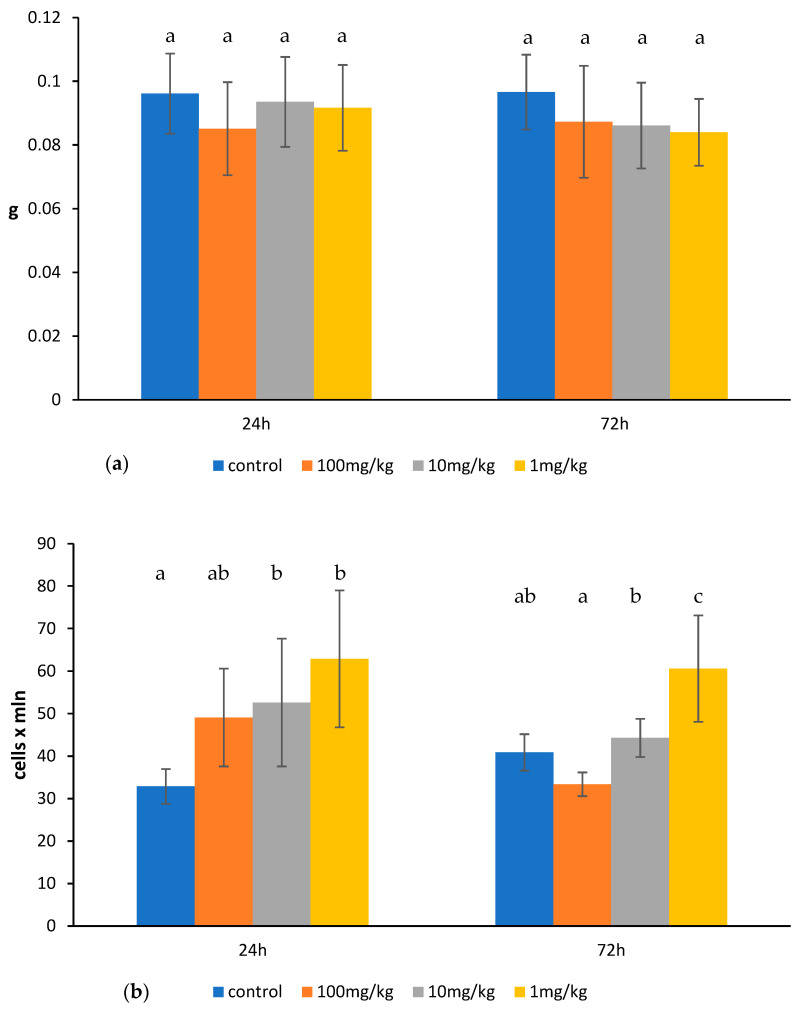

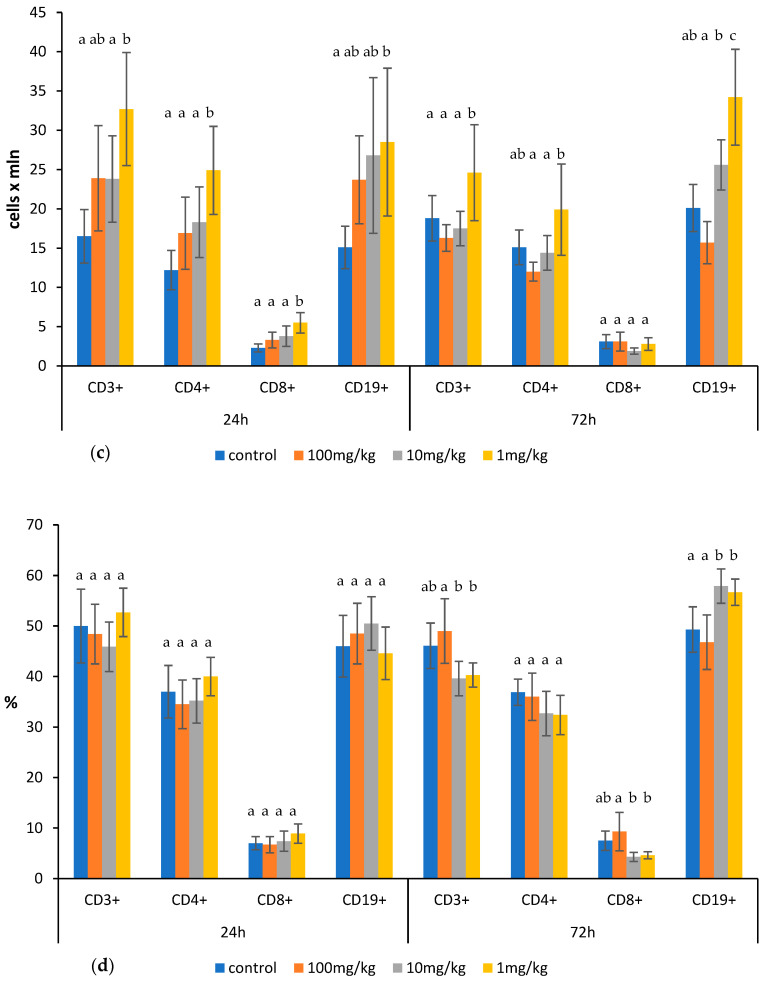
Mesenteric lymph node weight (**a**), total count of mesenteric lymph node lymphocytes (**b**), absolute count of mesenteric lymph node lymphocyte subpopulations (**c**), and the percentage of mesenteric lymph node lymphocyte subpopulations (**d**) in mice treated for 14 days (once a day) with ovophospholipids. The mean value (*n* = 7) and standard deviations are shown. Data with different superscript letters differ significantly (*p* < 0.05).

Ovophospholipids exerted a stimulating effect on the lymphocytes of the mesenteric lymph nodes. However, the effect was observed mostly for the lowest dose of 1 mg/kg. At that dose, ovophospholipids increased the total count of the lymphocytes of the mesenteric lymph nodes, which was observed 24 h (also at 10 mg/kg) and 72 h after the last administration. A rise in the absolute count of T lymphocytes (CD3^+^) with both CD4^+^ and CD8^+^ subpopulations and B lymphocytes (CD19^+^) was observed 24 h after the last administration. The effect on the absolute count of CD3^+^ and CD19^+^ lymphocytes was long-lasting, as it persisted 72 h after the last dose. Ovophospholipids administered at 10 and 1 mg/kg elevated also the percentage of CD19^+^ cells 72 h after the last administration (Figure 3b–d). Ovophospholipids at the examined doses did not affect mesenteric lymph node weight (Figure 3a).

### 2.2. Effect of Ovophospholipids on TNF-α and IL-1β Levels in the Culture Supernatants of Peritoneal Macrophages Stimulated In Vitro with Lipopolysaccharide (LPS) from E. coli (055:B5)

**Figure 4:**
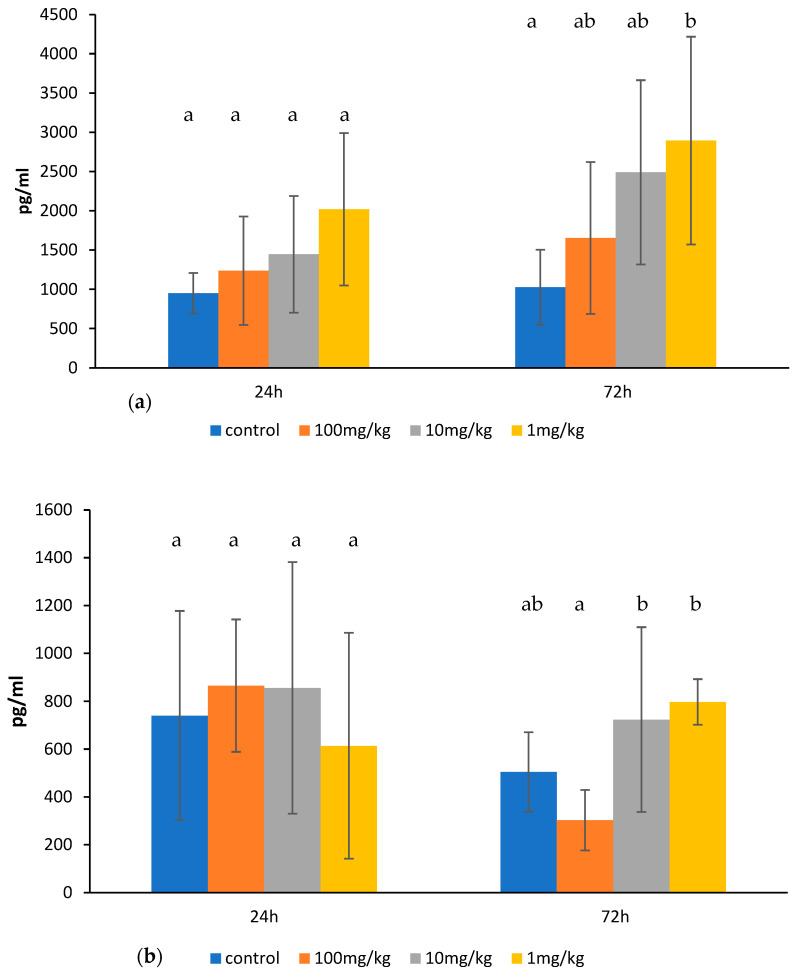
TNF-α (**a**) and IL-1β (**b**) levels in the culture supernatants of peritoneal macrophages obtained from mice treated with ovophospholipids for 14 days (once a day), stimulated in vitro with LPS. The mean value (*n* = 7) and standard deviations are shown. Data with different superscript letters differ significantly (*p* < 0.05).

An increase in TNF-α level was found for the ovophospholipid dose of 1 mg/kg 72 h after its last administration. However, an upward trend was also observed for other doses and also after 24 h (Figure 4a). Ovophospholipids at the examined doses did not affect IL-1β level (Figure 4b).

### 2.3. Effect of Ovophospholipids on Humoral Immune Response

**Figure 5:**
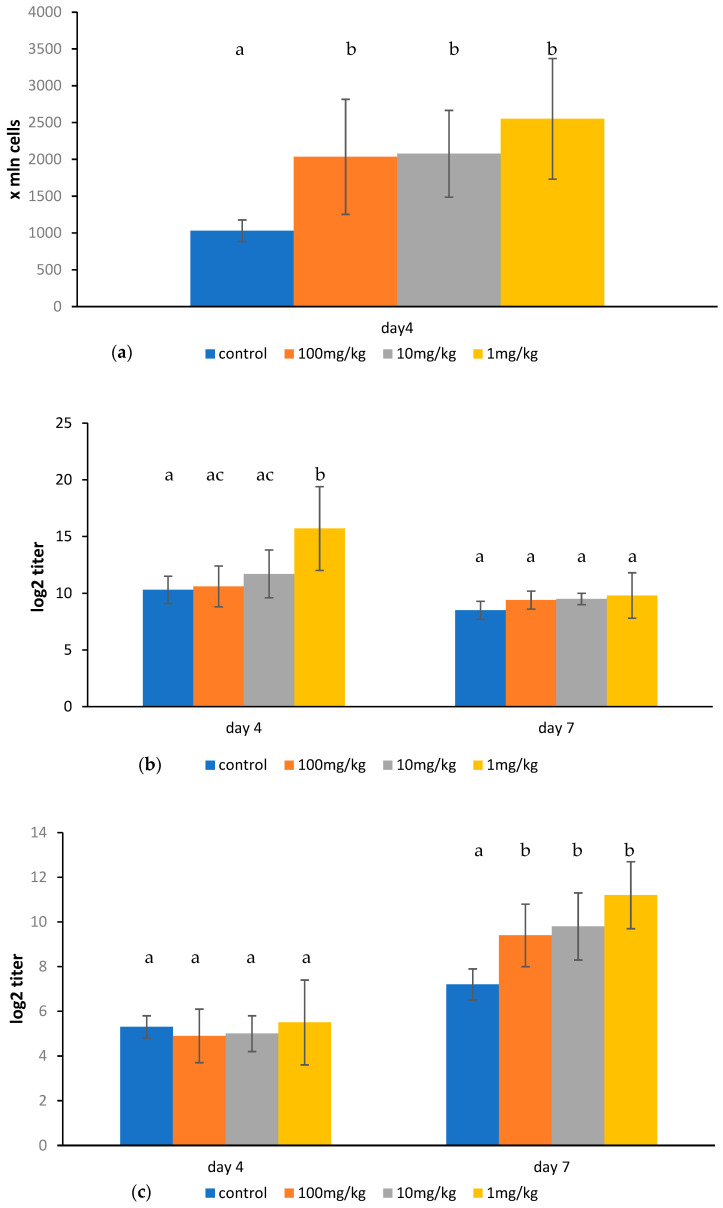
The number of PFCs: (**a**) total anti-SRBC hemagglutinin titer (**b**), and 2-ME-resistant anti-SRBC hemagglutinin titer (**c**) in SRBC-immunized mice treated for 14 days (once a day) with ovophospholipids. The mean value (*n* = 7) and standard deviations are shown. Data with different superscript letters differ significantly (*p* < 0.05).

Ovophospholipids administered for 14 days (once a day) elevated the number of plaque-forming cells (PFCs) on day 4 after SRBC immunization. The effect was observed for all examined doses. An increase in total anti-SRBC hemagglutinin titer was found only for the dose of 1 mg/kg on day 4 after SRBC injection. However, a stimulating impact of ovophospholipids on 2-ME-resistant antibody titer was noticed for all three doses on day 7 after immunization (Figure 5a–c).

## 3. Discussion

Studies on the biological activity of phospholipids and single fatty acids included in their composition have been conducted for many years. In the available literature, there are also numerous reports on the impact of different fatty acids on various aspects of the immune system. Ovophospholipids used in this study contain an enriched composition of fatty acids, especially n-3 PUFAs, i.e., DHA and EPA. In fact, no research has been published on the impact of ovophospholipids on the immune response. Therefore, in the discussion, we focused on the comparison of our results with the data from other authors regarding DHA/EPA or phospholipids of fish origin.

The impact of ovophospholipids on the lymphocytes in the thymus is ambiguous. An increase in the percentage and/or absolute count of immature CD4^+^CD8^+^ cells may suggest that ovophospholipids stimulate maturation of the thymic cells. However, the percentage of the mature CD4^+^ and CD8^+^ thymic cells was decreased. Taking into account that the population of T cells in the spleen and mesenteric lymph nodes increased, we may speculate that ovophospholipids accelerate migration of mature T cells from the thymus to the peripheral lymphoid organs.

The elevated total count of the thymus lymphocytes also observed in our study may be due to an increase in the number of immature thymocytes. In the available literature, there are almost no publications regarding the impact of fatty acids on thymocytes. The only study by Sasaki et al. [27] showed no effect on the number and percentage of thymocytes expressing CD3^+^, CD4^+^, and CD8^+^ in mice fed a diet varying in the amount of DHA for 4 weeks.

We noticed a stimulating effect of ovophospholipids on T lymphocytes of the peripheral lymphoid organs (spleen and mesenteric lymph nodes). Regarding the splenocytes, it was observed in particular 24 h after administration of the lower doses, i.e., 10 and 1 mg/kg. However, it seems that the impact of ovophospholipids on the splenocytes was rather short-lasting, as 72 h after the last administration most changes were not perceived anymore. Regarding mesenteric lymph nodes, ovophospholipids increased the population of T cells especially at the dose of 1 mg/kg.

The influence of phospholipids/n-3 PUFAs included in fish oil or purified DHA/EPA on the count and function of T lymphocytes was described by other authors. Similarly to our results, Sasaki et al. [27] found a significant increase in the number of splenocytes in mice fed for 4 weeks with DHA-rich diet. However, the surface expression of CD4^+^ and CD8^+^ on splenic T cells decreased as the dietary DHA concentration increased (the expression of CD3^+^ was unchanged). Analyzing the results of our team and those of Sasaki [27], we concluded that a certain regularity can be seen: the effect of phospholipids on the lymphocyte surface marker distribution strongly depends on the dose, and higher doses may affect the lymphocytes in different ways. In our study, the highest dose of ovophospholipids (100 mg/kg) had in fact no impact on T lymphocytes, contrary to the lower doses.

Other data [28] demonstrated that dietary fish oil enriched with DHA and administered for 12 weeks altered T lymphocyte populations and exacerbated colitis in mice. The percentage of CD4^+^CD3^+^ splenic T cells increased, but no differences were observed in the mesenteric lymph nodes. The percentage of CD8^+^CD3^+^ T lymphocytes was significantly reduced in both tissues. These results are in agreement with ours, as we also observed an increase in CD4^+^ splenocytes 24 h after the last administration of ovophospholipids and a decrease in the percentage of CD8^+^ splenocytes 72 h after the last administration.

A study by Paixão et al. [29] suggests that DHA and EPA had some anti-inflammatory activity and showed a beneficial effect on the immune system in newly diagnosed breast cancer patients. The anti-inflammatory effect was demonstrated by a constant level of serum high-sensitivity C-reactive protein (hsCRP) in DHA/EPA-treated patients, while in the placebo group, a significant increase in hsCRP was noticed. The immunomodulatory effect of the DHA/EPA administration was reflected in maintaining the percentage of peripheral blood CD4+ T cells, while in the placebo group, a significant reduction in the percentage of these cells was observed. The stimulating influence of ovophospholipids on CD4^+^ T lymphocytes of the peripheral lymphoid organs (spleen and mesenteric lymph nodes) found in our research corresponds with the finding of Paixão et al. [29], i.e., a lack of decrease in CD4+ cells, which in fact can also be considered as a stimulating effect of n3-PUFA on CD4+ lymphocytes.

We demonstrated a stimulating effect of ovophospholipids on B lymphocytes of the spleen and mesenteric lymph nodes. Most changes were observed after the administration of ovophospholipids at the lower doses of 10 and 1 mg/kg. The ovophospholipids administered at all examined doses also stimulated the humoral immune response.

These outcomes correlate with other studies. Teague et al. [30] showed that n-3 PUFAs administered for 4 weeks elevated the percentage and frequency of splenic B-cell subsets in the absence or presence of antigen in mice. Using a murine model of obesity, the researchers [30,31] also demonstrated the effect of a high-fat diet enriched with DHA or EPA (administered for 5 or 10 weeks) on B cell activity. DHA and EPA differentially raised spleen weight, the frequency, and/or percentage of selected B-cell subsets. The most prominent effect was noticed for the frequency and/or percentage of IgM^+^IgD^−^CD21^low^CD23^−^ B splenocytes. The total serum IgM level was elevated after feeding the animals with DHA and EPA, and cecal IgA increased following the EPA administration [30,31].

Another in vivo study [32] showed that a diet containing DHA-enriched fish oil (mice fed for 5 weeks) significantly raised the percentage of B cells (B220 used as a pan-B cell marker) and the expression of functional surface markers in the mesenteric lymph nodes and the Peyer’s Patches, as well as significantly elevated plasma levels of IL-5, IL-9, and cecal IgA.

IL-6 is involved in the maturation of B lymphocytes and stimulates B cells to produce immunoglobulins [33]. Gurzell et al. [32] reported that LPS-stimulated B cells from mice fed for 5 weeks with DHA-enriched fish oil significantly increased their CD40 expression and IL-6 production. Teague et al. [31] found that DHA or EPA (10-week supplementation) enhanced IL-6, TNFα, and IL-10 secretion by the murine B cells ex vivo. Thus, we can assume that the stimulating effect of ovophospholipids on B lymphocytes and humoral immune response is likely, at least in part, to be associated with their stimulating effect on the IL-6 production.

On the other hand, Weise et al. [34] showed in vitro that DHA inhibited, in a dose-dependent manner, the IgE production of human B cells by down-regulating both the CD40 and the IL-4 signaling pathway. The most prominent effect was observed for the highest concentration of DHA, i.e., 10 µM. The production of IgA was not altered, and the synthesis of IgG was only slightly reduced.

An explanation of this discrepancy in results may be the type of research (in vivo/ex vivo or in vitro). As found by Rockett et al. [35], the treatment of naïve B cells in vitro with 50 µM EPA or DHA for 48 h had no effect on the percentage of activated B cells, upregulation of cell surface molecules, and secretion of TNF-α, IFN-γ, and IL-10. Only the secretion of IL-6 was significantly suppressed. Ex vivo, when mice were fed n-3 PUFAs diet, there was still no effect on the percentage of B cells activated by LPS; however, this diet increased the B-cell CD69 surface expression and IL-6 and IFN-γ secretion [35].

We noticed a stimulating effect of the ovophospholipids administered at the dose of 1 mg/kg on the synthesis of TNF-α by murine peritoneal macrophages, and an upward trend was also observed for other doses. However, no changes were found in the level of IL-1β.

The influence of n3-PUFA on these cytokines was reported by other authors [6,7,8,31]. However, the results are inconsistent. Gorjao et al. [6] also demonstrated an increase in the synthesis of TNF-α by lymphocytes derived from male volunteers consuming DHA- and EPA- enriched fish oil. Contrary to that, Kew et al. [7] reported that supplementation of human diet with a DHA-rich oil had no effect on cytokine (i.a. TNF-α and IL-1β) production by peripheral blood mononuclear cells. Interestingly, Tan et al. [8] demonstrated that administration of EPA + DHA to aging adults with chronic venous leg ulcers significantly reduced high levels of circulating proinflammatory cytokines, i.e., IL-6, IL-1β, and TNF-α. Taking into account the findings presented above, we may conclude that the impact of n3-PUFA on the cytokines depends on many factors. The experimental protocol is one of the key determinants. We estimated the level of TNF-α and IL-1β produced by peritoneal macrophages stimulated in vitro with LPS while the other authors assessed these parameters in blood samples.

Stimulation of macrophages by LPS reflects infection and induction of inflammation in the body. TNF-α is one of the agents whose concentration increases in such situations, and one of its roles is the stimulation of cytotoxic activity of monocytes and macrophages as a response to, i.a., infection. Therefore, ovophospholipid-induced enhancement in TNF-α production by macrophages may contribute to more efficient response to infections. On the other hand, a rapid release of large amounts of TNF-α is highly unfavorable and can lead to respiratory and organ failure. Since we stimulated macrophages in vitro, further studies are needed to clearly assess the importance of the ovophospholipids in the TNF-α production.

Another fact worth discussing is that TNF-α induces the release of some cytokines, i.a., IL-6; and together with this cytokine may enhance proliferation and differentiation of B lymphocytes. Therefore, we cannot exclude the possibility that the stimulating effect of ovophospholipids on TNF-α synthesis may also indirectly contribute to the stimulating effect of ovophospholipids on B lymphocytes and humoral immune response.

Some differences between our findings and those of other authors may be attributed to several factors. One of the postulated mechanisms by which phospholipids influence immune cells is that PUFAs are incorporated into the cellular membranes and thus alter the lipid composition of the membrane microdomains and receptor signaling function [36]. Therefore, the composition of phospholipids, i.e., quantitative and qualitative content of individual fatty acids as well as their final administered dose/concentration are important factors determining the final result of the experiment. Moreover, differences in the time of exposure, which may also reflect the age and the metabolic profile of an animal, and the baseline immune status could influence the final effect. Other factors may include differences in experimental protocols and the type of experiment—in vitro versus ex vivo/in vivo study.

This work demonstrated that lower doses of ovophospholipids had more pronounced effect on the determined parameters. It is not surprising, as in our previous studies on other immunomodulating agents, we also found stronger immunomodulatory effects after the administration of lower experimental doses compared to higher ones [37,38].

In conclusion, our research showed that ovophospholipids exert a stimulating effect on the lymphocyte subsets and the humoral immune response in mice. Most changes were observed after the administration of the ovophospholipids at the lower doses of 10 and 1 mg/kg. Regarding T lymphocytes, the effect is rather short-lived, as most changes were observed only after 24 h following the administration of the last dose.

Stimulation of lymphocytes and immunoglobulin production are the pillars of the cellular and humoral immune response, respectively. Therefore, we may assume that the administration of ovophospholipids, resulting in an increase in lymphocyte subsets and antibody titers, may contribute to better resistance against infections or diseases. However, as there are many inconsistencies in the literature regarding the impact of fatty acids on the immune response, more experiments with ovophospholipids, taking into consideration different aspects of the immune system, should be carried out to assess their complex immunomodulatory potential and possible clinical indications.

## 4. Materials and Methods

### 4.1. Animals

The experiments were conducted on female Balb/c mice, aged 8–10 weeks, and with body weight of 18–20 g. The mice were purchased from the Breeding Centre of Laboratory Animals at the Institute of Occupational Medicine (Łódź, Poland). They were kept under conventional conditions and fed commercial food and water ad libitum. The study was conducted according to the requirements of the European Union, with the consent of the II Local Ethics Committee for Animal Experiments, Wrocław, Poland (permission number 12/2019).

### 4.2. Drugs and Treatments

Ovophospholipids were obtained in the project: “Innovative technologies of the production of biopreparations based on the new generation of eggs (OVOCURA)”. Laying hens (Lohmann Brown line) were fed ad libitum with standard feed supplemented with fish oil (3%), dried algae (1.5%), Humokarbowit and Humobentofet humic preparations, and vitamin E (0.01%). The details of the method of ovophospholipid isolation were described in the Patent application No. 218452. The composition of the ovophospholipids is presented in Table 1.

Ovophospholipids were dissolved in aqueous starch solution and administered to mice orally (via a stomach tube) at the doses of 100, 10, and 1 mg/kg, for 14 days at 24 h intervals. The dose volume was 0.2 mL per mouse. The experiments in the control group were conducted simultaneously. The control mice received pure aqueous starch solution without the preparation. Each experimental and control group consisted of seven mice.

### 4.3. Measurements

The studies were performed according to two experimental protocols—in non-immunized mice and SRBC-immunized mice.

The following parameters were assessed in the non-immunized mice: (1) the weight of the thymi, spleens, and mesenteric lymph nodes; (2) the total number of lymphocytes in the thymus, spleen, and mesenteric lymph nodes; (3) the percentage and absolute count of lymphocyte subsets in the lymphatic organs, i.e., CD4^−^CD8^−^, CD4^+^CD8^+^, CD4^+^, and CD8^+^ in the thymus; CD19^+^, CD3^+^, CD4^+^, and CD8^+^ in the spleen and mesenteric lymph nodes; (4) synthesis of TNF-α and IL-1β in the culture supernatants of peritoneal macrophages stimulated in vitro with LPS from *E. coli* (055:B5). The parameters were assessed 24 h and 72 h after administration of the last dose of ovophospholipids. The absolute counts were calculated based on the total count of lymphocytes in individual lymphatic organs.

In the second experimental protocol, the mice were immunized with 4 × 10^8^ SRBC per mouse (0.2 mL of 10% SRBC suspension), administered intraperitoneally 24 h after the last dose of ovophospholipids. Sheep blood was collected into Alsever’s solution in sterile manner and kept at 4 °C for at least 3 days. The SRBC suspension in phosphate-buffered saline (PBS, Institute of Immunology and Experimental Therapy, Wrocław, Poland) was prepared ex tempore.

The following parameters in the SRBC-immunized mice were evaluated: (1) the number of PFCs in the spleen, and (2) anti-SRBC hemagglutinin titer in the serum. The number of PFCs was determined on day 4 after priming, while the number of anti-SRBC hemagglutinin titer was assessed on days 4 and 7 after immunization.

### 4.4. Determination of Total Count and Subsets of Lymphocytes in the Thymus, Spleen, and Mesenteric Lymph Nodes

The mice were anesthetized with halothane (Narcotan, Zentiva, Prague, Czech Republic) and killed by cervical dislocation. The thymuses, spleens, and mesenteric lymph nodes were weighed and placed into Petri dishes with sterile, ice-cold PBS. The suspended cells were obtained by passing the lymphatic organs through a nylon mesh into 1 mL of PBS. The number of lymphocytes in the thymus, spleen, and mesenteric lymph nodes was counted with a Thoma hemocytometer. The cell suspension was centrifuged (3000× *g*, 15 min, 4 °C) on a layer of Ficoll 400 (Pharmacia, Fine Chemicals AB, Uppsala, Sweden)/Urografin 76% (diatrizoate sodium and meglumine diatrizoate, Bayer Schering Pharma, Poland) in a 1:3 ratio, density of 1.076. Next, the lymphocytes were collected from the interphase and washed twice (375× *g*, 7 min, 4 °C) with ice-cold PBS supplemented with 1% bovine serum albumin (BSA; Sigma, Saint Louis, MO, USA). Lysis of erythrocytes present in the splenocyte suspensions was induced by incubation of the splenocytes with 0.84% ammonium chloride (POCH, Gliwice, Poland) for 5 min at 37 °C. After the second wash, the cells were resuspended in PBS with 1% BSA at 1 × 10^7^ cells/mL. Viability of each cell suspension was assessed using the trypan blue dye exclusion assay. It was above 95%. Then, the thymocytes, splenocytes, and lymphocytes from the mesenteric lymph nodes resuspended in PBS containing 1% BSA were stained with the monoclonal Rat Anti-Mouse CD4:FITC/CD8:RPE dual-color reagent (AbD Serotec, Kidlington, UK) at a dilution rate recommended by the manufacturer. The lymphocytes from the spleen and mesenteric lymph nodes were stained additionally with the monoclonal Rat Anti-Mouse CD19:FITC/CD3:RPE dual-color reagent (AbD Serotec, Kidlington, UK) according to the manufacturer’s instructions. The cells were incubated with the antibodies for 30 min at 4 °C and washed three times (375× *g*, 7 min, 4 °C) with ice-cold PBS. Fluorescence was analyzed with a flow cytometer (FACS Calibur; Becton-Dickinson Biosciences, San Jose, CA, USA). Distribution of the thymocyte, splenocyte, and lymphocyte of the mesenteric lymph node markers was analyzed with CellQuest 3.1 f. Pro software (Becton-Dickinson Biosciences, San Jose, CA, USA).

### 4.5. Determination of Synthesis of TNF-α and IL-1β in the Culture Supernatants of Peritoneal Macrophages Stimulated In Vitro with LPS from E. coli (055:B5)

Mice were anesthetized with halothane and killed by cervical dislocation. Peritoneal macrophages were harvested by lavage of the peritoneal cavity with sterile, ice-cold PBS supplemented with antibiotics: penicillin 10 U/mL and streptomycin 10 μg/mL (a stabilized Penicillin–Streptomycin solution, Sigma). The cells were centrifuged (375× *g*, 10 min, 4 °C). Then, the macrophages were suspended in RPMI-1640 medium (Institute of Immunology and Experimental Therapy, Wrocław, Poland) supplemented with 10% fetal bovine serum (FBS, Sigma), 10 mM HEPES (Sigma), 2 mM L-glutamine (Sigma), 10 U/mL of penicillin and 10 μg/mL of streptomycin. Viability of the macrophages was determined by the trypan blue dye exclusion assay, and it was 90–95%. Purity of the peritoneal cell suspension was estimated with the Pappenheim method. The macrophages accounted for 65–75% of the whole cell population. The macrophages were washed twice and adjusted to a concentration of 1.5 × 10^6^ cells/mL. The cells were dispensed in a 96-well flat bottom plate (Nunc, Roskilde, Denmark) and incubated 3 h at 37 °C with 5% CO_2_. After this time, the medium was replaced, and the incubation was continued for 18 h at the same conditions. Then, the medium was replaced again with the medium without FBS but with LPS from *E. coli*, serotype 055:B5 (Sigma) at a concentration of 2.5 μg/mL. The incubation was continued for further 24 h. Then, the supernatants were removed and stored at −80 °C.

Murine IL-1β and TNF-α levels in the culture supernatants of peritoneal macrophages were determined with commercial ELISA kits (Quantikine Mouse IL-1β and TNF-α Immunoassays, R&D Systems, Minneapolis, MN, USA) according to the manufacturer’s protocols. The results were read on a spectrophotometric microplate reader (Elx800, BioTek, Winooski, VT, USA) at 450 nm with the wavelength correction of 570 with KC Junior software 1.4.

### 4.6. Determination of Plaque Forming Cells

The mice were euthanized by cervical dislocation after previous anesthesia with halothane. The spleens were removed and placed into ice-cold Hank’s saline (Institute of Immunology and Experimental Therapy, Wrocław, Poland). The splenocytes were isolated according to the lymphocyte subsets’ assay. After isolation, the cells were collected from the interphase and washed twice in Hank’s saline (375× *g*, 7 min, 4 °C). Next, the cells were resuspended in Hank’s saline to a concentration of 1 × 10^6^ cells/mL. Viability of the splenocytes was determined by trypan blue dye exclusion assay, and it was above 95%. To assess the number of splenocytes producing hemolytic anti-SRBC antibodies (PFCs), a local hemolysis technique in an agar gel was used [39].

### 4.7. Determination of Serum Anti-SRBC Antibodies

Blood was collected from the retro-ocular arteries of the halothane-anesthetized mice and centrifuged for 15 min at 3500× *g*. Then, the sera were inactivated at 56 °C for 30 min. To determine the total (IgM + IgG) and 2-mercaptoethanol (2-ME)-resistant (IgG) serum hemagglutinin titers, an active hemagglutination test, carried out on microplates, was used [40]. The results were expressed as a value of log_2_.

### 4.8. Statistical Analysis

The data obtained in the study were analyzed statistically using a one-way analysis of variance (ANOVA), followed by Tukey’s post hoc test for multiple comparisons. The differences were considered significant at *p* < 0.05.

## Figures and Tables

**Table 1:** Fatty acid composition in ovophospholipids.

Acetone Insoluble Matter 75.68% Phosphatidylcholine (Lecithin) 75.6% Phosphatidylethanolamine (Cephalin) 24.4%	Amount (%)
Acids	
C14:0	0.53
C14:1	0.16
C16:0	25.51
C16:1	2.69
C17:0	0.19
C18:0	14.48
C18:1	27.09
C18:2; ω–6	13.43
C18:3; ω–3	3.32
C20:2; ω–6	0.16
C20:3; ω–6	0.30
C20:4; ω–6	1.99
C20:5; ω–3	0.92
C22:6; ω–3	9.24
Sum of ω–3 fatty acids	13.48
Sum of ω–6 fatty acids	15.88
ω–6/ω–3	1.18
Total saturated fatty acids (SFAs)	40.71
Total unsaturated fatty acids (UFAs)	59.29
Total MUFAs	29.94
Total PUFAs	29.35

## Data Availability

The original contributions presented in this study are included in the article material. Further inquiries can be directed to the corresponding author.

## References

[1] Bubel F., Dobrzański Z., Bykowski P., Patkowska-Sokoła B., Trziszka T. (2011). Enrichment of hen eggs with omega-3 polyunsaturated fatty acids-physiological and nutritional aspects. Acta Sci. Pol. Med. Weterinaria.

[2] Sun N., Chen J., Wang D., Lin S. (2018). Advance in food-derived phospholipids: Sources, molecular species and structure as well as their biological activities. Trends Food Sci. Technol..

[3] Anton M., Huopalahti R., López-Fandiño R., Anton M., Schade R. (2007). Composition and Structure of Hen Egg Yolk. Bioactive Egg Compounds.

[4] Xu D., Ren J., Ali B., Jin Y., Jin Z., Xu X. (2021). Water-in-oil soybean concentrated phospholipids hydrolysis based on the model of enzymatic deactivation and its application in bread. Food Biosci..

[5] Cherian G. (2008). Egg quality and yolk polyunsaturated fatty acid status in relation to broiler breeder hen age and dietary n-3 oils. Poult. Sci..

[6] Gorjão R., Verlengia R., Lima T.M., Soriano F.G., Boaventura M.F., Kanunfre C.C., Peres C.M., Sampaio S.C., Otton R., Folador A. (2006). Effect of docosahexaenoic acid-rich fish oil supplementation on human leukocyte function. Clin. Nutr..

[7] Kew S., Mesa M.D., Tricon S., Buckley R., Minihane A.M., Yaqoob P. (2004). Effects of oils rich in eicosapentaenoic and docosahexaenoic acids on immune cell composition and function in healthy humans. Am. J. Clin. Nutr..

[8] Tan A., Sullenbarger B., Prakash R., McDaniel J.C. (2018). Supplementation with eicosapentaenoic acid and docosahexaenoic acid reduces high levels of circulating proinflammatory cytokines in aging adults: A randomized, controlled study. Prostaglandins Leukot. Essent. Fat. Acids.

[9] Olson M.V., Liu Y.C., Dangi B., Paul Zimmer J., Salem N., Nauroth J.M. (2013). Docosahexaenoic acid reduces inflammation and joint destruction in mice with collagen-induced arthritis. Inflamm. Res..

[10] Che H., Li H., Song L., Dong X., Yang X., Zhang T., Wang Y., Xie W. (2021). Orally Administered DHA-Enriched Phospholipids and DHA-Enriched Triglyceride Relieve Oxidative Stress, Improve Intestinal Barrier, Modulate Inflammatory Cytokine and Gut Microbiota, and Meliorate Inflammatory Responses in the Brain in Dextran Sodium Sulfate Induced Colitis in Mice. Mol. Nutr. Food Res..

[11] Merzouk S.A., Saker M., Reguig K.B., Soulimane N., Merzouk H., Guermouche B., Berrouiguet A.Y., Hichami A., Narce M., Khan N.A. (2008). N-3 polyunsaturated fatty acids modulate in-vitro T cell function in type I diabetic patients. Lipids.

[12] Chapkin R.S., Arrington J.L., Apanasovich T.V., Carroll R.J., McMurray D.N. (2002). Dietary n-3 PUFA affect TcR-mediated activation of purified murine T cells and accessory cell function in co-cultures. Clin Exp Immunol..

[13] Kong W., Yen J.H., Ganea D. (2011). Docosahexaenoic acid prevents dendritic cell maturation, inhibits antigen-specific Th1/Th17 differentiation and suppresses experimental autoimmune encephalomyelitis. Brain Behav. Immun..

[14] Lian M., Luo W., Sui Y., Li Z., Hua J. (2015). Dietary n-3 PUFA Protects Mice from Con A Induced Liver Injury by Modulating Regulatory T Cells and PPAR-γ Expression. PLoS ONE.

[15] Han S.C., Koo D.H., Kang N.J., Yoon W.J., Kang G.J., Kang H.K., Yoo E.S. (2015). Docosahexaenoic Acid Alleviates Atopic Dermatitis by Generating Tregs and IL-10/TGF-β-Modified Macrophages via a TGF-β-Dependent Mechanism. J. Investig. Dermatol..

[16] Carlsson J.A., Wold A.E., Sandberg A.S., Östman S.M. (2015). The Polyunsaturated Fatty Acids Arachidonic Acid and Docosahexaenoic Acid Induce Mouse Dendritic Cells Maturation but Reduce T-Cell Responses In Vitro. PLoS ONE.

[17] Monk J.M., Hou T.Y., Turk H.F., McMurray D.N., Chapkin R.S. (2013). n3 PUFAs reduce mouse CD4^+^ T-cell ex vivo polarization into Th17 cells. J. Nutr..

[18] Teague H., Rockett B.D., Harris M., Brown D.A., Shaikh S.R. (2013). Dendritic cell activation, phagocytosis and CD69 expression on cognate T cells are suppressed by n-3 long-chain polyunsaturated fatty acids. Immunology.

[19] Vedin I., Cederholm T., Freund Levi Y., Basun H., Garlind A., Faxén Irving G., Jönhagen M.E., Vessby B., Wahlund L.O., Palmblad J. (2008). Effects of docosahexaenoic acid-rich n-3 fatty acid supplementation on cytokine release from blood mononuclear leukocytes: The OmegAD study. Am. J. Clin. Nutr..

[20] Rusnak T., Azarcoya-Barrera J., Makarowski A., Jacobs R.L., Richard C. (2024). Plant- and Animal-Derived Dietary Sources of Phosphatidylcholine Have Differential Effects on Immune Function in The Context of A High-Fat Diet in Male Wistar Rats. J. Nutr..

[21] Yessoufou A., Plé A., Moutairou K., Hichami A., Khan N.A. (2009). Docosahexaenoic acid reduces suppressive and migratory functions of CD4+CD25+ regulatory T-cells. J. Lipid Res..

[22] Wu S., Peng H., Li S., Huang L., Wang X., Li Y., Liu Y., Xiong P., Yang Q., Tian K. (2024). The ω-3 Polyunsaturated Fatty Acid Docosahexaenoic Acid Enhances NK-Cell Antitumor Effector Functions. Cancer Immunol. Res..

[23] Gutiérrez S., Svahn S.L., Johansson M.E. (2019). Effects of Omega-3 Fatty Acids on Immune Cells. Int. J. Mol. Sci..

[24] Miles E.A., Calder P.C. (2012). Influence of marine n-3 polyunsaturated fatty acids on immune function and a systematic review of their effects on clinical outcomes in rheumatoid arthritis. Br. J. Nutr..

[25] Tomasdottir V., Thorleifsdottir S., Vikingsson A., Hardardottir I., Freysdottir J. (2014). Dietary omega-3 fatty acids enhance the B1 but not the B2 cell immune response in mice with antigen-induced peritonitis. J. Nutr. Biochem..

[26] Juman S., Hashimoto M., Katakura M., Inoue T., Tanabe Y., Arita M., Miki T., Shido O. (2013). Effects of long-term oral administration of arachidonic acid and docosahexaenoic acid on the immune functions of young rats. Nutrients.

[27] Sasaki T., Kanke Y., Kudoh K., Misawa Y., Shimizu J., Takita T. (1999). Effects of dietary docosahexaenoic acid on surface molecules involved in T cell proliferation. Biochim. Biophys. Acta.

[28] Woodworth H.L., McCaskey S.J., Duriancik D.M., Clinthorne J.F., Langohr I.M., Gardner E.M., Fenton J.I. (2010). Dietary fish oil alters T lymphocyte cell populations and exacerbates disease in a mouse model of inflammatory colitis. Cancer Res..

[29] Paixão E.M.D.S., Oliveira A.C.M., Pizato N., Muniz-Junqueira M.I., Magalhães K.G., Nakano E.Y., Ito M.K. (2017). The effects of EPA and DHA enriched fish oil on nutritional and immunological markers of treatment naïve breast cancer patients: A randomized double-blind controlled trial. Nutr. J..

[30] Teague H., Fhaner C.J., Harris M., Duriancik D.M., Reid G.E., Shaikh S.R. (2013). n-3 PUFAs enhance the frequency of murine B-cell subsets and restore the impairment of antibody production to a T-independent antigen in obesity. J. Lipid Res..

[31] Teague H., Harris M., Fenton J., Lallemand P., Shewchuk B.M., Shaikh S.R. (2014). Eicosapentaenoic and docosahexaenoic acid ethyl esters differentially enhance B-cell activity in murine obesity. J. Lipid Res..

[32] Gurzell E.A., Teague H., Harris M., Clinthorne J., Shaikh S.R., Fenton J.I. (2013). DHA-enriched fish oil targets B cell lipid microdomains and enhances ex vivo and in vivo B cell function. J. Leukoc. Biol..

[33] Kaur S., Bansal Y., Kumar R., Bansal G. (2020). A panoramic review of IL-6: Structure, pathophysiological roles and inhibitors. Bioorg Med. Chem..

[34] Weise C., Hilt K., Milovanovic M., Ernst D., Rühl R., Worm M. (2011). Inhibition of IgE production by docosahexaenoic acid is mediated by direct interference with STAT6 and NFκB pathway in human B cells. J. Nutr. Biochem..

[35] Rockett B.D., Salameh M., Carraway K., Morrison K., Shaikh S.R. (2010). n-3 PUFA improves fatty acid composition, prevents palmitate-induced apoptosis, and differentially modifies B cell cytokine secretion in vitro and ex vivo. J. Lipid Res..

[36] Fan Y.Y., Fuentes N.R., Hou T.Y., Barhoumi R., Li X.C., Deutz N.E.P., Engelen M.P.K.J., McMurray D.N., Chapkin R.S. (2018). Remodelling of primary human CD4+ T cell plasma membrane order by n-3 PUFA. Br. J. Nutr..

[37] Jine Y., Lis M., Szczypka M., Obmińska-Mrukowicz B. (2012). Influence of betulinic acid on lymphocyte subsets and humoral immune response in mice. Pol. J. Vet. Sci..

[38] Lis M., Szczypka M., Suszko A., Obmińska-Mrukowicz B. (2011). Influence of bestatin, an inhibitor of aminopeptidases, on T and B lymphocyte subsets in mice. Pol. J. Vet. Sci..

[39] Mishell R.I., Dutton R.W. (1967). Immunization of dissociated spleen cell cultures from normal mice. J. Exp. Med..

[40] Hudson L., Hay F.C. (1980). Practical Immunology.

